# Magnetic Iron Oxide Nanoparticles as T_2_ MR Imaging Contrast Agent for Detection of Breast Cancer (MCF-7) Cell

**Published:** 2017

**Authors:** Pegah Moradi Khaniabadi, Daryoush Shahbazi-Gahrouei, Mohammad Suhaimi Jaafar, Amin Malik Shah Abdul Majid, Bita Moradi Khaniabadi, Saghar Shahbazi-Gahrouei

**Affiliations:** 1. Faculty of Physics, University Sains Malaysia, Pulau Penang, Malaysia; 2. Department of Medical Physics, Faculty of Medicine, Isfahan University of Medical Sciences, Isfahan, Iran; 3. Faculty of Medicine, University Sains Malaysia, Penang, Malaysia; 4. Child Growth and Development Research Center, Research Institute for Primordial Prevention of Non-communicable Disease, Isfahan University of Medical Sciences, Isfahan, Iran; 5. Faculty of Medicine, Isfahan University of Medical Sciences, Isfahan, Iran

**Keywords:** Breast cancer, Contrast media, Magnetic resonance imaging, Nanoparticles

## Abstract

**Background:**

Advances of nanotechnology have led to the development of nano-materials with both potential diagnostic and therapeutic applications. Among them, Super Paramagnetic Iron Oxide Nanoparticles (SPIONs) have received particular attention. Modified EDC coupling fraction was used to fabricate the SPION-C595 as an MR imaging contrast agent for breast cancer detection in early stages.

**Methods:**

Nanoprobe characterization was confirmed using Fourier Transform Infrared Spectroscopy (FT-IR), Scanning Electron Microscopy with Energy Dispersive X-Ray Spectroscopy (SEM-EDAX), and Photon Correlation Spectroscopy (PCS). Protein and iron concentration of nanoprobe was examined by standard method. MTT assay was performed to evaluate the cytotoxicity of the nanoprobe in breast cancer cell line (MCF-7). T_2_-weighted MR imaging was performed to evaluate the signal enhancement on T_2_ relaxation time of nanoprobe using spin-echo pulse sequence.

**Results:**

As results showed, SPIONs-C595 provided active targeting of breast cancer cell (MCF-7) at a final concentration of 600 *μgFe/ml*. The final concentration of protein was calculated to be at 0.78 *μgprotein/ml*. The hydrodynamic size of the nanoprobe was 87.4±0.7 *nm*. The MR imaging results showed a good reduction of T_2_ relaxation rates for the highest dose of SPIONs-C595.

**Discussion:**

Based on the results, SPIONs-C595 nanoprobe has a potential in T_2_-weighted MR imaging contrast agent for breast cancer cell (MCF-7) detection.

## Introduction

Recently, nanotechnology has turned out to be a fundamental word of public attention. Among all kinds of nanoparticles, biocompatible Superparamagnetic Iron Oxide Nanoparticles (SPIONs) with appropriate surface as well as structure were considered as molecular imaging and drug delivery tools ^1–3^. SPIONs have an outstanding function as greatly sensitive molecular-specific imaging nanoprobes ^4^. Magnetic Fe_3_O_4_ is a very common type of iron oxide, which has advantages including high magnetization ^5^, hyperthermia ^6^ together with biocompatibility for *in vivo* as well as *in vitro* features. Iron oxide nanoparticle compounds identified by the nanosized diameter and their structure allow different biodistribution and possibility of imaging chemical agents ^7^.

Nanomedicine plays an essential role by delivering the contrast agent in a targeted manner to specific tumor cells, leading to improvement in accurate diagnosis by good visualization and specific demonstration of tumor cells. Effective and specific diagnostic imaging of breast cancer in early stages is a major challenge ^8,9^.

By taking advantage of nanoparticles as contrast agents, potential of MR imaging might be extended to diagnosis of tumors in the early stages ^10^. The main purpose of using contrast agents is to enhance signal intensity which is the fact of shortening relaxation times. Diagnostic imaging of tumors using mabs has previously been investigated ^11,12^. High molecular weight glycoproteins are usually identified as mucins or mucin-like glycoproteins. One of the targets at breast tumor is breast specific membrane antigen (MUC1). The C595 mabs has been considered as a reagent of clinical applicability and utilized in immunoassays for determination of circulating mucin in individuals with breast cancer. C595 mabs contains immunogenicity due to its murine origin in addition to poor tumor penetration characteristics due to large molecular size (150 *kDa*) ^13^.

Among molecular imaging techniques, the MR imaging provides very high spatial resolution along with soft tissue contrast. With the use of specific molecular contrast agents, MR imaging allows imaging on anatomic-morphologic and molecular ranges with a single method. In particular, SPIONs are perfectly useful for cell imaging because they promote strong susceptibility effects that make it possible for sensitive detections of even small amounts of cellular material. In MR imaging, contrast is generated by modulation of water relaxivity (inverse of T_1_ and T_2_ relaxation times) through its chemical environment or through the introduction of contrast agents that modulates the local magnetic field ^14^.

One of the new approaches is to investigate the increases of the specificity of MR image contrast agents by using mab C595 coupled with SPIONs. In the research by Liu *et al*, the contrast agent (C225-USPION) which was the result of conjugating Ultra SPION and cetuximab for detecting the epithelial growth factor receptors was introduced ^15^. Binding USPION with C595 mab, in order to detect *in vivo* MUC1 expression, was studied by Shanehsazzadeh *et al* ^16^. They used a dual contrast agent, C595 antibody-conjugated USPION labeled with ^99m^Tc as the target of anti-MUC1-expressing cancers for imaging and therapy purposes. Shahbazi-Gahrouei *et al* studied on developing ovarian cancer (OVCAR3) by C595 mab which was bounded to SPIONs. The strategy was using SPIONs attached to C595 mAb that bound to the MUC1, in order to detect ovarian cancer cells by using MR imaging ^12^.

This study investigated the fabrication, characterization and application of a specific breast cancer MR imaging contrast agent, C595-mab conjugated SPIONs (SPIONs-C595) by simplified EDC method, for breast cancer detection with MUC1 over expression in early stages as an MR imaging contrast agent.

## Materials and Methods

All chemicals were purchased by Sigma (USA) and were used without further purification. Nanomag-D-spio 20 *nm* nanoparticles (surface COOH) as well as miniMACS separator in addition to C595 mab were purchased from Miltenyi Biotech GmHb, Germany. The breast cancer cell line, (MCF-7) EA.hy926 cell as control was obtained from ATCC, USA. MCF-7 and EA.hy926 cells were routinely cultured in pre-warmed DMEM (37*°C* in water bath) supplemented with 10% of Fetal Bovine Serum (FBS), antibiotics (100 *IU/ml* penicillin and 100 *μg/ml* streptomycin), 1% *v/v* essential amino acids and 2 *mM* L-glutamine and used for further procedure.

### Conjugation of mab C595 with SPIONs

**Figure 1.**
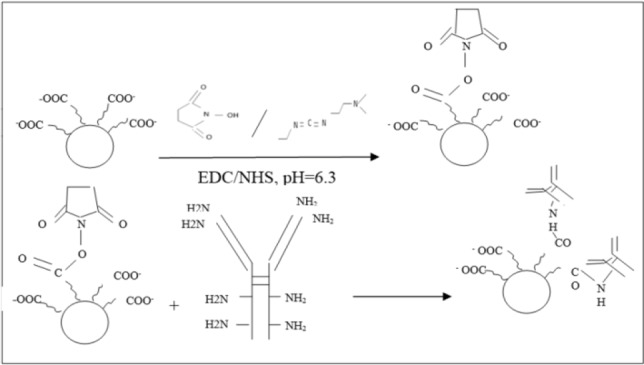
Bioconjugation scheme for SPIONs-C595s by using EDC.

A 500 *μl* of the nanomag®-D-spio (Miltenyi Biotech GmbH, Germany) was mixed with working solution. The working solution was prepared by adding 0.6 *mg* (3 *μmol*) N-ethyl-N-(3-dimethyl aminopropyl) carbodiimide hydrochloride (EDC) and 1.2 *mg* (10 *μmol*) N- hydroxyl Succinimide (NHS) in 125 *μl* 0.5 *M* 2-(N-morpholino) ethanesulfonic acid (MES) buffer according to the Gruttner *et al* ^17^. For neutral pH reaction, a phosphate buffer at 0.5 *M* was appropriate. The suspension was mixed for 90 *min* at room temperature by a shaker. Then, suspension was washed twice with 1 *ml* Phosphate Buffered Saline (PBS), pH=7.4 through MS column. To activate the columns before adding mixture, the columns were washed three times with PBS, pH=7.4, each time. Then, 100 *μl* C595 mab was added for nanoparticles activation and the suspension was shaken for 3 *hr*. After 3 *hr*, the suspension was washed two times with 1 *ml* PBS through MS column. In this step, unconjugated antibodies were separated from conjugated ones. Figure 1 shows chemical schematic of this reaction. As seen in this figure, only when the carboxyl groups of the SPION were activated, the C595 mab attached to the SPION surface ^18^.

### Surface chemistry

A 1 *ml* bulk nanomag®-D-spio solution and SPIONs-C595 were frozen in 15 *ml* falcon tube at −20*°C* for 12 *hr* at −18*°C* (Elba EF-3500, Italy). The samples were then freeze-dried under vacuum at −50*°C* for 48 *hr* using a freeze-drying system (Labconco FreeZone 6, USA). Transmission FTIR spectra were recorded from thin KBr disc of the samples with Perkin Elmer 2000 FTIR spectrophotometer at room temperature (25*°C*). The samples were scanned from 4000 to 400 *cm*^−1^, and the range of wavelength was in infrared zone. All the spectra were collected after an average of 16 scans for each specimen. The analysis was done automatically by software implemented at School of Chemical Sciences, USM.

### Visualization of the morphology of SPIONs-C595

Scanning Electron Microscopy with Energy Dispersive X-Ray Spectroscopy (SEM/EDAX) was used to study the surface morphology of the samples, which included nanomag®-D-spio dried in an oven by 60*°C* over night and SPIONs-C595 freeze-dried as mentioned earlier. Samples were fixed onto the specimen holder by using double face adhesive tape and were coated with Cadmium using Quorum sputter coater (Q150T ES, UK). The samples were then examined under a scanning electron microscope (Quanta FEG 560, USA) located at the Centre for Global Archaeological Research, USM. Images were captured under conventional secondary electron imaging conditions with an acceleration voltage of 10 *kV* and at different magnifications of 100, 500, and 3000 folds. The samples were vacuumed for 5 to 10 *min* before analysis.

### Particle size and PCS

Particle size, Polydispersity Index (PDI) and zeta potential were determined by photon correlation spectroscopy (PCS) using a Zetasizer nano zs (Malvern Instruments Ltd, UK). The two nanomag®-D-spio and SPIONs-C595 suspensions were diluted in DIW and MES buffer, respectively. The samples were mixed well by pipette in order to obtain a uniform suspension to avoid any agglomeration. Prior to measurement, the samples were filtered through 0.2 *nm* syringe filter to separate any visible particles in the samples. Analysis was performed in INFORMM, USM.

### Iron and protein concentration measurement

Iron concentration of SPIONs-C595 nanoprobe was obtained by Potassium Thiocyanate method ^18^. Briefly, to get the calibration curve to measure the concentration of the finalized compound, Iron atomic spectroscopy standard, 1 *g* Fe (Sigma-Aldrich) was used. Before preparing the series of different concentrations of the standard solution, the Potassium Thiocyanate (KSCN) solution as well as 6N HNO_3_ containing 1% H_2_O_2_ was prepared in a 50 *ml* volumetric flask. For KSCN solution, 5 *g* of KSCN (SYSTERM) was weighed with balance (Fisher Scientific, USA). The chemical was then dissolved in Deionised Water (DIW) up to the mark of 50 *ml* volumetric flask at room temperature. KSCN reacted with DIW, and therefore gave a deep red coloration to solution containing Fe^3+^. Then, 300 *μl* of the 6N HNO_3_ containing 1% H_2_O_2_ solution was pipetted into wells to make the total volume of 1 *ml* in each well. The iron was detected at 405 *nm* in the assay using a microplate reader (Infinite@200 PRO, TECAN, Switzerland). The absorbance of the standard samples and SPION-C595 samples were measured. As long as the volume of the standard samples and the compound samples were the same, final concentration of the SPION-C595 was directly calculated from the least squares regression line of standard curve.

The amount of immobilised antibodies in SPION-C595 was estimated based on the Quick Start^TM^ Bradford protein assay ^19^. The assay was based on the theory that when protein binds, the pKa of the dye shifts causing the dye to become blue.

### Cell viability

MCF-7 cells were grown in culture flasks with media (15 *ml* media/175 *cm*^3^) and incubated at 37*°C*, in a humidified atmosphere of 95%/5% air/CO_2_. The methylthiazolydiphenyl-tetrazolium bromide (MTT) reagent was freshly prepared at 5 *mg/ml* in a sterile PBS, filtered through 0.2 *μm* syringe filter and further diluted in a fresh culture medium to obtain a final concentration of 600 *μg/ml*. A 20 *μl* of MTT solution was added to the cold culture medium in each well, and 200 *μl* of culture medium containing the MTT reagent was incubated for 3 to 5 *hr*. At the end of the incubation, the supernant was aspirated carefully and the water insoluble formazan salt was solublized in 100 *μl* DMSO per well. After 10 *min* incubation at 37*°C*, optical density of the violet was measured by a microplate reader at a primary wavelength of 570 *nm* and a reference wavelength of 620 *nm*. The cell viability was calculated by applying the following formula:

$$\text{Cell}\;\text{viability}=({\text{OD}}_{\text{Samples}}-{\text{OD}}_{\text{Blank}})/{\text{OD}}_{\text{Negative}\;\text{control}}-{\text{OD}}_{\text{Blank}})$$

The percentage inhibition was calculated as:

%Inhibition=(1−cell viability)×100%

### MR imaging protocol

MR imaging was performed by Signa HDxt 1.5 T, Optima at IPPT, USM. A matrix size of 256×128 was used for data reconstruction by 2-D Fourier transformation. The samples were placed at the center of the standard circular polarized head coil. The field of view was 20 *mm*. The slice thickness and number of slices were 3 without distance between the slices.

To determine T_2_ relaxivity (R_2_), the T_2_ relaxation times of six concentrations (200, 100, 50, 25, 12.5, 6.25 *μg Fe/ml*) of each D-SPION and SPIONs-C595 were measured and their signal intensity was extracted by Image J software. Distilled water was used as the control for all studied samples. The SPIONs were diluted in distilled water and the SPION-C595 was diluted by 0.9 normal saline solutions. Images were obtained using T_2_-weighted images method with T_R_= 2000 *ms* and T_E_=12, 24, 36 and 48 *ms*. The measurement of signal intensity was performed on the images using region of interest (ROI) with a constant size for all echoes. Magnetic resonance signal intensities were driven by the following equation^4^:

$$\text{SI}={\text{M}}_{0}({\text{e}}^{-\text{TE}/\text{T}2})(1-{\text{e}}^{-\text{TR}/\text{T}1})$$

when the T_2_ relaxation rate of the superparamagnetic nanoparticles increases, its ability to shorten the proton relaxation time becomes stronger. Relaxivity was also calculated by plotting 1/T_2_ over concentration and determining the slope of the regression line.

## Results

### Surface chemistry

**Figure 2.**
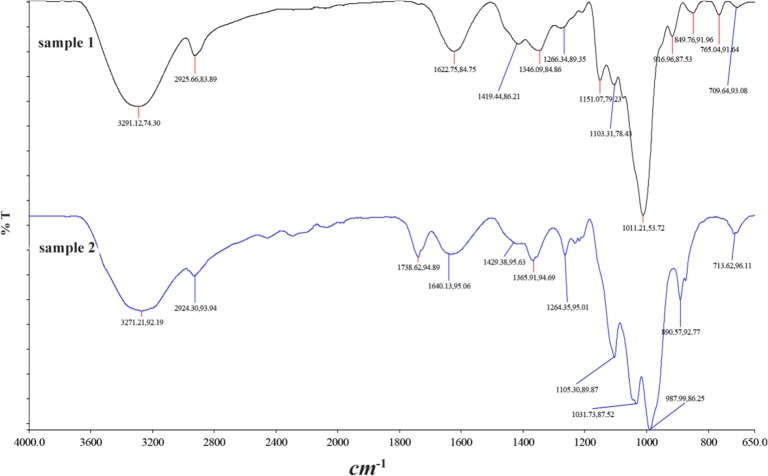
FT-IR result of freeze dried nanomag®-D-spio (sample 1) and SPION-C595 (sample 2).

The FT-IR spectra of nanomag®-D-spio (sample 1) and SPIONs-C595 (sample 2) were presented in figure 2 which demonstrated the conjugation of C595 mab with the iron oxide nanoparticles. Carboxylic acids show a strong wide band at the O-H stretch in the region of 3300–2500 *cm*^−1^. The broad band was shown in the IR spectrum of nanomag-D-spio, even though it became broader after conjugation because the O-H stretch band of carboxylic acids exists as hydrogen-bonded dimers. The broad band at 3291 and 3271 *cm*^−1^ corresponds to the O-H groups in the dicarboxylic acid structure.

The characteristic peak corresponding to the stretching vibration of the Fe-O bond shifted to a higher wave numbers of 713 *cm*^−1^ (sample 2), compared to 709 *cm*^−1^ (sample 1) reported for the stretching of the Fe-O in bulk Fe_3_O_4_ after conjugating with C595 mab ^10^.

The carbonyl stretch C=O of carboxylic acids appeared as an intense band from 1760–1690 *cm*^−1^. However, according to figure 2, the conversion of the COOH group to EDC collapses the carbonyl band to a simpler structure at about 1738 *cm*^−1^, typical of an electron-deficient ester carbonyl. The C-O stretch appeared alongside it in the region of 1320–1210 *cm*^−1^. The -CH_2_ stretching vibrations presented on SPION-C595 (sample 2) were seen to increase intensity following the attachment of the mab onto the nanoparticle. The amine group stretching vibrations at 1105 and 1031 *cm*^−1^ also increased in absorbance in sample 2.

In the IR spectra of proteins, the secondary structure is most clearly reflected by the amide I and amide II bands, particularly the former, which had absorption of around 1620 to 1690 *cm*^−1^ and is primarily associated with the stretching vibrations of peptide carbonyl groups ^20^. Evidence for the half-amide/ester structure (sample 2) was found in the FT-IR spectrum in which both the ester and the amide carbonyl peaks were clearly seen at 1738 *cm*^−1^ which confirmed the successful surface modification.

### SEM and EDAX

**Figure 3.**
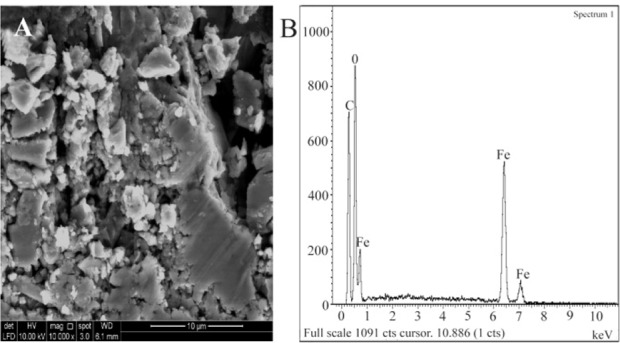
A) SEM morphology of the nanomag®-D-spio and B) EDAX spectrum of nanomag®-D-spio in black line.

**Figure 4.**
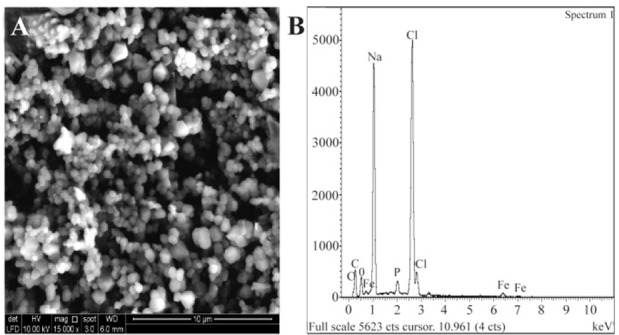
A) SEM morphology of the SPIONs-C595 and B) EDAX spectrum of SPIONs-C595 in black line.

Results of the SEM for nanomag®-D-spio and SPIONs-C595 samples are shown in figures 3 and 4. Figure 3A represents the morphology and figure 3B represents the EDAX spectrum of nanomag®-D-spio. Elemental analysis of nanomag®-D-spio revealed the presence of iron and oxygen atoms, in a percentage that indicates iron oxides. Figure 4A shows the SPIONs-C595 morphology of nanoparticles with round and smooth surfaces, while figure 4B shows the elemental analysis of nanoprobe. EDAX results suggested the presence of iron and oxygen elements, which conforms to the presentation of iron after conjugation with C595 antibodies ^21^.

### Particle size and PCS

The size of nanoparticle as well as the surface charge of contrast agents after conjugation with C595 mab was measured. The spherical shape, average particle size, and the width of the particle size distribution (polydispersity) was estimated by Dynamic Light Scanning (DLS) and results are presented in table 1 and it shows the z-average hydrodynamic diameter of nanomag®-D-spio and SPIONs-C595. Also, the size of NP was reported to be 51.3±0.07 *nm* with a polydispersity index of 0.2. The z-average hydrodynamic diameter of NP increased after conjugation with C595 mab to 87.4±0.7 *nm* with a polydispersity index of 0.3, which was still an ideal size for application in transfection.

**Table 1.** Analysis of particle size and zeta potential of nanomag®-D-spio and SPIONs-C595. Results are displayed as AV±SD (n=2)

	**Particle diameter (*nm*)**	**Mean intensity (*nm*)**	**Count rate (*kCps*)**	**Polydispersity index (PDI)**	**Volume mean (*nm*)**	**Zeta potential (*mV*)**
**Nanomag®-D-spio**	51.3±0.1	59.6±0.1	253.9±0.3	0.20±0.1	34.1	−28.9±1.0
**SPION-C595**	87.4±0.7	131.8±8.9	91.1±8.4	0.30±0.1	71.4	0.262±0.1

### Iron and protein concentration determination

**Figure 5.**
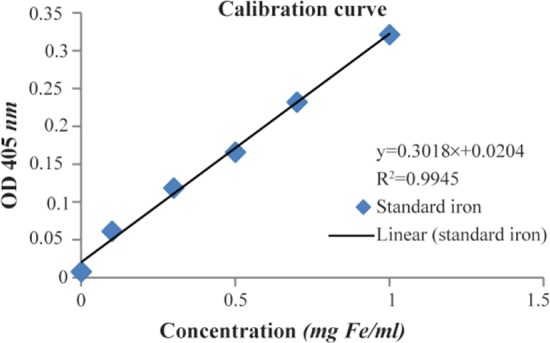
Standard curve of Iron standard concentration (*μgFe/ml*) versus absorbance at 405 *nm*.

**Figure 6.**
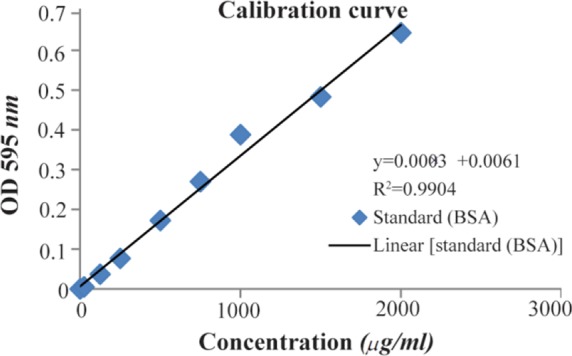
The standard curve of BSA concentration measurements. UV-visible spectroscopy measurement was carried out for known concentration of BSA at the absorbance maximum of 595 *nm*

The amount of iron in the SPION-C595 nanoprobe was estimated using the Potassium Thiocyanate method. The final iron concentration of the nanoprobe was directly calculated from the least squares line of the standard curve (Figure 5). The calibration curve was plotted according to the absorbance of different concentrations of standard iron versus the samples as an unknown sample. The final iron concentration of the SPION-C595 nanoprobe was 600 *μgFe/ml*. The final antibody concentrations of the SPION-C595 nanoprobe were directly calculated from the least squares line of the standard curve (Figure 6). The R2 was calculated as 0.9904 (which was close to 1). The final antibody concentration of the SPION-C595 nanoprobe was 0.78 *μg protein/ml*.

### SPIONs-C595 Uptake

**Figure 7.**
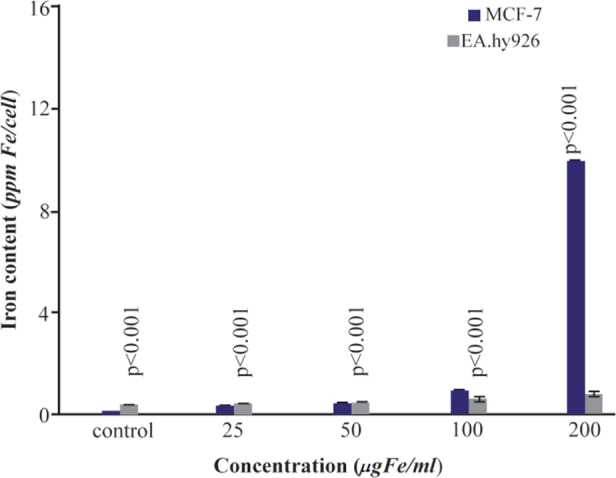
Iron uptake in MCF-7 and EA.hy926 cells. Cells were incubated with 25–200 *μgFe/ml* of SPIONs-C595s at 37 °C for 6 *hr*

The cellular uptake of the SPIONs-C595 nanoprobe was verified on cancerous (MCF-7) and normal (EA. hy926) cell lines. Atomic Absorption Spectroscopy (AAS) results of the same concentrations of SPION-C595 were applied; both cell lines are shown in figure 7. This figure shows the iron content of each cell lines after 6 *hr* of incubation with different concentrations of SPION-C595 measured using the AAS technique. At lower concentrations (25 and 50 *μgFe/ml*), the iron content in EA.hy926 and MCF-7 was the same. 100 *μg Fe/ml* of nanoprobe caused the iron content in MCF-7 to increase, while at 200 *μgFe/ml*, the iron content of SPIONs-C595 in MCF-7 was significantly higher than that in EA.hy926. The results shows that, by increasing the concentration of the compound, the possibility of attachment of the nanoprobe to the breast cancer cells is increased compared to normal cells.

### Cell viability

**Figure 8.**
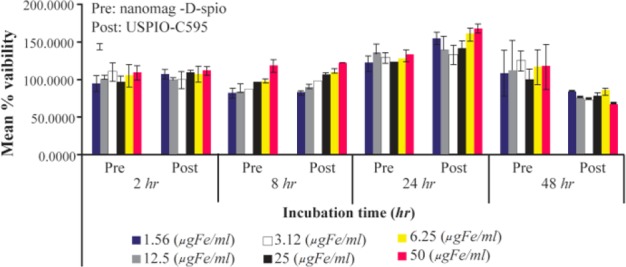
Viability of MCF-7 cells exposed to different concentrations (6.25–100 *μgFe/ml*) of Nanomag®-D-spio and SPIONs-C595 for different time points, 2 to 48 *hr*.

To assess any potential cytotoxicity of these SPIONs towards the cells, they were exposed to different SPION concentrations (1.56, 3.12, 6.25, 12.50, 25.00, 50.00 *μgFe/ml*) for 2, 8, 24, and 48 *hr* and analyzed by MTT assays for cell viability and results are shown as ±SD (Figure 8). As seen from this figure, there was no cytotoxicity upon conjugation, even at the highest concentration of components.

### T_2-_weighted MR imaging

**Figure 9.**
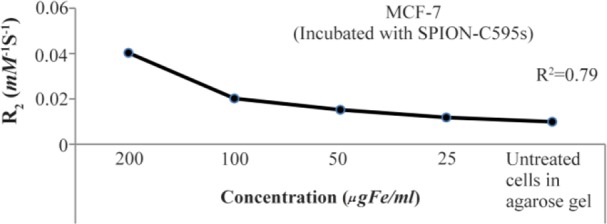
The graph of R_2_ versus different concentrations of SPIONs-C595 after 6 *hr* of incubation with MCF-7 cells

To evaluate the T_2_ enhancing capability of the SPIONs-C595, nanoparticles before and after conjugation were imaged with different concentrations using T_2_ spin-echo pulse sequence and the base was (1% agarose gel) to reduce the noise of background during imaging protocol and results are presented in table 2 and figure 9. As the results show, the magnetic resonance contrast enhancement of SPIONs-C595 in MCF-7 was evaluated with various concentrations of contrast agent and a good linear correlation between R_2_ and the iron concentration was established. When the T_2_ relaxation rate of the studied nanoparticle increases, its ability to shorten the proton relaxation time increases.

**Table 2.** T_2_ relaxation time and relaxivity (R_2_) values of MCF-7 cells incubated with different concentrations of SPIONs-C595 incubated with MCF-7 after 6 *hr*

		**R_2_ (*mM*^−1^*sec*^−1^)**	**T_2_ (*ms*)**
**Concentration (*μgFe/ml*)**			
	200	0.0403	24.81
	100	0.0202	49.50
	50	0.0152	65.78
	25	0.0118	84.74
**Agarose gel**		0.0094	106.38
**Water**		0.0220	454.54
**Untreated cells in agarose gel**		0.0099	101.01

In addition, the reduction of the spin-spin relaxation times (T_2_) was observed from the lowest to highest concentrations, in which the signal intensity of the T_2_-weighted images decreased. The negative (T_2_) contrast agents are so-called because they reduce magnetic relaxation times which results in a hyperintense change of resonance signal in MR imaging. This observation showed that T_2_-weighted signal intensities of SPION-C595s treated MCF-7 cell were darker than those untreated EA.hy926 cells as the control for all Fe concentrations used ^22^. The reductions in magnetic relaxation of water protons in the presence of SPION-C595 are caused by the very strong relaxation of spins in an inhomogeneous magnetic field which gives rise to the magnetic nuclei of the SPIONs conjugated C595 mab ^4,11^.

## Discussion

Surface chemistry modification of SPION-C595 is critical to ensure that the conjugation of the nanoparticles and C595 mabs. Evidence for the half-amide/ester structure was found in the FT-IR spectrum in which both the ester and the amide carbonyl peaks were clearly seen at 1738 and 1640 *cm*^−1^ which confirmed the successful surface modification. In addition, the IR spectra of protein, the secondary structure is most clearly reflected by the amide I and amide II bands, which have absorption of around 1620 to 1690 *cm*^−1^. It was reported that the magnetic nanoparticle surface modification can increase protein absorption and assist the nanoparticles uptake in cancer cells.

EDAX results suggested the presence of iron and oxygen elements, which conforms to the presentation of iron after conjugation with C595 antibodies ^21^. The increase in hydrodynamic size of the nanoparticle after conjugation was about 30 *nm*, thus, indicating antibody conjugation with the nanoparticle. The polydispersity value stood at 0.3, indicates that the nanoparticle suspension was almost homogenous, in terms of the biomedical and bioengineering applications of any nanoparticle including iron oxide, the size must be smaller than 100 *nm* with overall narrow particle size distribution, so that the particles have uniform physical and chemical properties ^22^.

By the EDC method obtained in this research, the amine group was added to improve the chance of attachment of the C595 mab onto the surface of nanoparticles. As a result, the surface charge of nanoparticle reduced after conjugation. Therefore, the cell phagocytosis rate of nanoparticle with positively-charged surface was faster that the nanoparticle with neutral or negatively-charged surfaces. Due to the fact that nanoparticles with positively-charged surface are able to adhere more easily to the negatively-charged surface of cell membranes by electrostatic interaction.

Iron belongs to a group of elements known as transition metals, as it is essential element for the survival of almost all organisms that live in an oxygen-rich environment. It is required for many metabolic processes such as oxygen transport, drug metabolism, cellular respiration, electron transport, and cell proliferation. Thus, according to the results of cellular uptake (Figure 7), iron was detected in the control sample. Therefore, at lower concentration of SPIONs-C595 after incubation with normal cells. The results of SPIONs-C595 uptake by the cells shows that, by increasing the concentration of the compound, the possibility of attachment of the nanoprobe to the breast cancer cells is increased compared to normal cells. The results suggest that compared to the breast cancer cells, there is no significant cellular uptake by the normal cells after administration of a higher SPIONs-C595 dose. As can be seen from figure 9, the magnetic resonance contrast enhancement of SPIONs-C595 in MCF-7 was evaluated with various concentrations of contrast agent and a good linear correlation between R_2_ and the iron concentration was established. When the T_2_ relaxation rate of the studied nanoparticle increases, its ability to shorten the proton relaxation time increases ^11^.

The T_2_ values refer to the speed of magnetization in the plan perpendicular to the static magnetic field loose coherence. The results of T_2_-weighted images showed the reduction of the spin-spin relaxation times (T_2_) from the lowest to highest concentrations, in which the signal intensity of the T_2_-weighted images decreased. The negative (T_2_) contrast agents are so-called because they reduce magnetic relaxation times which results in a hyperintense change of resonance signal in MR imaging. This observation showed that T_2_-weighted signal intensities of SPIONs-C595 treated MCF-7 cell were darker than those untreated EA.hy926 cells as the control for all Fe concentrations used ^23^. The reductions in magnetic relaxation of water protons in the presence of SPIONs-C595 are caused by the very strong relaxation of spins in an inhomogeneous magnetic field which gives rise to the magnetic nuclei of the SPIONs conjugated C595 mab ^4,11^. The diffusion of water molecules around the magnetic centres leads to the partial averaging of local magnetic fields experienced by a spin during MR imaging ^24^.

## Conclusion

The use of SPIONs-C595 nanoprobe was demonstrated via several characterization techniques, such as FT-IR, SEM-EDAX, Zetasizer and protein and iron concentration measurements. As the results showed, SPIONs-C595 provided active targeting of breast cancer cell (MCF-7) at a final concentration of 600 *μgFe/ml*. Moreover, the final concentration of protein was calculated to be at 0.78 *μgprotein/ml*. The hydrodynamic size of the nanoprobe was 87.4±0.7 *nm*, 30 *nm* more than the actual size of the SPIONs due to the increase in the size from the attachment of C595 mab to SPIONs. The MR imaging results showed a good reduction of T_2_ relaxation rates for the highest dose of SPIONs-C595. Based on the results of the present study, SPIONs-C595 nanoprobe has a potential in T_2_-weighted MR imaging contrast agent for breast cancer (MCF-7) cell detection.
